# Belhassen Ventricular Tachycardia in a Child

**DOI:** 10.19102/icrm.2021.120207

**Published:** 2021-02-15

**Authors:** Brojendra Agarwala, Frank J. Zimmerman

**Affiliations:** ^1^Department of Pediatrics, Pediatric Cardiology, University of Chicago Medicine and Advocate Children’s Heart Institute, Oak Lawn, IL, USA

**Keywords:** Belhassen tachycardia, right bundle branch block, verapamil

## Abstract

First reported in 1981, idiopathic left ventricular tachycardia (VT) of the Belhassen type is characterized during electrocardiography (ECG) by a right bundle branch pattern and left axis deviation. We report the case of a 15-year-old Hispanic male who, during a routine evaluation ECG to support sports participation, was found to have nonsustained monomorphic VT. Prior to his exercise treadmill test, his physical examination and echocardiogram were normal. Then, during preparation for the exercise treadmill test, the ECG showed sustained monomorphic VT with a right bundle branch block pattern and superior QRS axis, suggesting a diagnosis of Belhassen VT.

## Introduction

Idiopathic left ventricular tachycardia (VT) of the Belhassen type originates from the left posterior fascicle and, less commonly, from the left anterior fascicle. First reported by Belhassen in 1981,^1^ it is characterized during electrocardiography (ECG) by a right bundle branch pattern and left axis deviation.^2^

**Figure 1:**
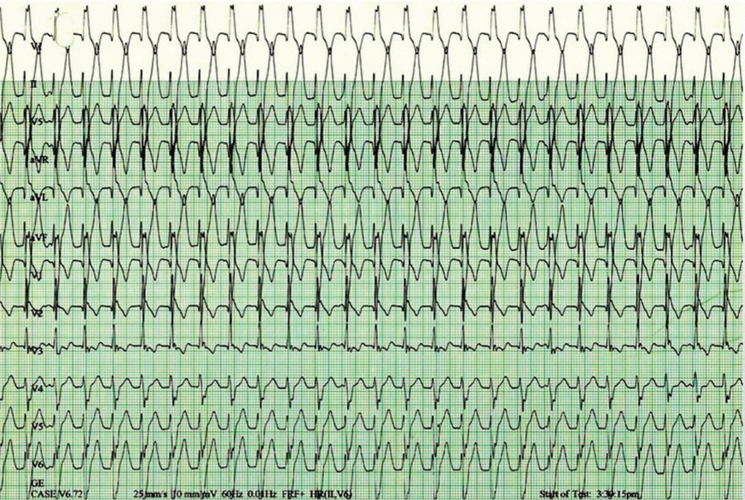
ECG taken during preparation for the exercise treadmill test showing VT with left axis deviation and RBBB.

The purpose of this report is to describe the management of a 15-year-old Hispanic male who, during a routine evaluation ECG to clear him for sports participation, was found to have a nonsustained monomorphic VT. Prior to his exercise treadmill test, his physical examination and echocardiogram were normal. However, during preparation for the exercise treadmill test, an ECG showed sustained monomorphic VT with a right bundle branch block (RBBB) pattern and superior QRS axis, suggesting a diagnosis of Belhassen VT **(Figure 1)**.

## Case presentation

A 15-year old Hispanic male was noted to have nonsustained monomorphic VT on a routine school ECG conducted to clear him for sports participation. The QRS morphology during a nonsustained VT was RBBB pattern with a superior axis. He was asymptomatic at the time of presentation. During a review of further history, he complained of palpitations, chest pain, and one episode of syncope. His past medical history, review of systems, and family history were unremarkable. A physical examination revealed an overweight young man in no distress. His vital signs were: weight of 258 pounds, height of 161 cm, heart rate of 97 bpm, respiratory rate of 18 breaths/min, and blood pressure of 118/72 mmHg. Cardiac examination revealed a quiet precordium without any palpable heave or thrill and both the first and second heart sounds were normal, with no audible murmur or gallop. Peripheral pulses were normal and equal in the upper and lower extremities. The rest of his physical examination was normal and his echocardiogram was normal.

**Figure 2:**
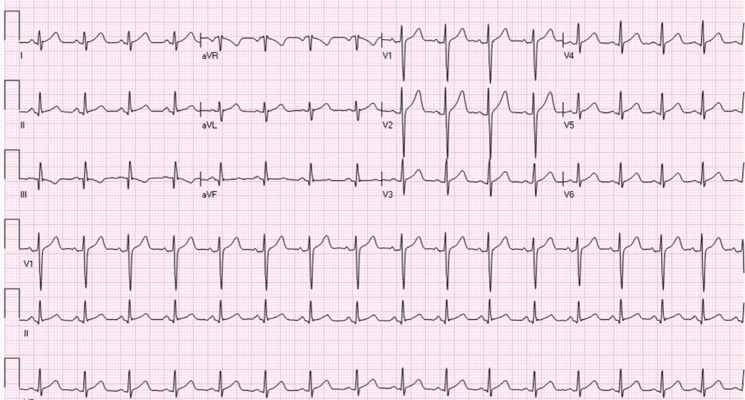
ECG recorded after radiofrequency ablation showing normal sinus rhythm and normal QRS axis.

During preparation for the exercise treadmill test, however, his ECG showed sustained monomorphic VT with RBBB pattern and superior QRS axis, heart rate of 142 bpm, and QRS duration of 125 ms **(Figure 1)**. At this point, the exercise test was discontinued and he was sent to the emergency room. The VT was converted to sinus rhythm with intravenous verapamil. He was not started on chronic medications but underwent an electrophysiology (EP) study and was found to have to have idiopathic left VT arising from the left posterior fascicle. The left posterior fascicle was first identified during sinus rhythm by mapping of the Purkinje potentials. VT was induced with ventricular extrastimulus testing from the right ventricular (RV) apex. Entrainment of the VT was not performed but has been described; in particular, manifest ventricular entrainment has been described with pacing from the RV outflow tract.^3^ Activation mapping was then performed during VT. The site of earliest ventricular activation (corresponding to the VT exit site) was seen near the infero-apical portion of the LV septum. Purkinje potential mapping was then performed during tachycardia. There was a retrograde pattern (apical to base) seen in the Purkinje potentials. Late diastolic potentials were not recorded. The earliest Purkinje potentials were targeted for ablation. This site was more basal than the infero-apical exit site seen with activation mapping and radiofrequency ablation here terminated the tachycardia. There was no change in the QRS duration or frontal axis following ablation **(Figure 2)**. The patient remained asymptomatic and not on any medications at two years of follow-up.

## Discussion

Sustained monomorphic VT in young patients is often associated with congenital heart defects, mostly in the postoperative period; ischemic heart disease; or myocardial dysfunction. VT occurring in a structurally normal heart with no apparent cause is labeled as “idiopathic” and arises from the RV or LV outflow tracts in 90% of cases. In 10% to 15% of cases, however, idiopathic VT can originate from near the LV apex involving the left septal intrafascicular system (ie, idiopathic left VT of the Belhassen type).^4^ Detailed EP studies have suggested the mechanism of this tachycardia is due to a reentry circuit usually involving the left posterior fascicle and adjacent abnormal Purkinje tissue with slow, decremental calcium-dependent conduction. The entrance site of the slow conduction zone is thought to be near the base of the LV septum, while the exit site is more apical. During tachycardia, the retrograde limb consists of Purkinje tissue from the left posterior fascicle. The anterograde limb is composed of abnormal Purkinje tissue adjacent to the left posterior fascicle that boasts slow, decremental conduction that is verapamil-sensitive and insulated from the surrounding myocardium. Less common sites of reentry include the left anterior fascicle or the left upper septal fascicle.^3^ Another cause of idiopathic left VT involves automaticity from the Purkinje fibers. The ECG of this tachycardia usually shows an RBBB with a superior axis; however, the QRS duration is wider and there is no rsR’ pattern in the lead V1 when compared with in the context of fascicular VT.^3^ The findings on the ECG and during the EP study in this case suggested the existence of reentry using the left posterior fascicle.

Patients with Belhassen VT are usually young and healthy, with the first episode of VT occurring in adolescence.^5^ Common clinical symptoms are palpitations, dizziness, fatigue, and shortness of breath; however, they are usually hemodynamically stable.^5,6^ The tachycardia may be precipitated by exercise, excitement, or infection.^5^ The typical ECG characteristics of idiopathic left VT using the left posterior fascicle are monomorphic VT with RBBB morphology, superior axis, and a QRS duration of 120 ms to 140 ms.^7^ The tachycardia is sensitive to intravenous verapamil in 90% of cases and, rarely, to adenosine.^2,3,7^ Chronic therapy regimens with β-blockers, oral verapamil, and class 1 or class 3 antiarrhythmic agents have been described.^3,4,7^ Treatment with radiofrequency ablation has been reported with overall success rates of 70% to 90%.^3–5^ Indications for ablation include sustained tachycardia associated with LV dysfunction, hemodynamic compromise, or symptomatic tachycardia as an alternative to medical treatment.^8^ In the present case, ablation was performed based on patient choice as an alternative to chronic medical management.
